# Shape Perception and Navigation in Blind Adults

**DOI:** 10.3389/fpsyg.2017.00010

**Published:** 2017-01-17

**Authors:** Monica Gori, Giulia Cappagli, Gabriel Baud-Bovy, Sara Finocchietti

**Affiliations:** ^1^Unit for Visually Impaired People, Istituto Italiano di TecnologiaGenoa, Italy; ^2^Robotics, Brain and Cognitive Science Department, Istituto Italiano di TecnologiaGenoa, Italy; ^3^The Unit of Experimental Psychology, Division of Neuroscience, IRCCS San Raffaele Scientific Institute, Vita-Salute San Raffaele UniversityMilan, Italy

**Keywords:** shape, perception, audio perception, blindness, motor, navigation

## Abstract

Different sensory systems interact to generate a representation of space and to navigate. Vision plays a critical role in the representation of space development. During navigation, vision is integrated with auditory and mobility cues. In blind individuals, visual experience is not available and navigation therefore lacks this important sensory signal. In blind individuals, compensatory mechanisms can be adopted to improve spatial and navigation skills. On the other hand, the limitations of these compensatory mechanisms are not completely clear. Both enhanced and impaired reliance on auditory cues in blind individuals have been reported. Here, we develop a new paradigm to test both auditory perception and navigation skills in blind and sighted individuals and to investigate the effect that visual experience has on the ability to reproduce simple and complex paths. During the navigation task, early blind, late blind and sighted individuals were required first to listen to an audio shape and then to recognize and reproduce it by walking. After each audio shape was presented, a static sound was played and the participants were asked to reach it. Movements were recorded with a motion tracking system. Our results show three main impairments specific to early blind individuals. The first is the tendency to compress the shapes reproduced during navigation. The second is the difficulty to recognize complex audio stimuli, and finally, the third is the difficulty in reproducing the desired shape: early blind participants occasionally reported perceiving a square but they actually reproduced a circle during the navigation task. We discuss these results in terms of compromised spatial reference frames due to lack of visual input during the early period of development.

## Introduction

While navigating, sighted individuals rely on both visual and non-visual sensory information. Multisensory integration plays a critical role in the representation of space development. Sensory information converges onto multisensory areas of the parietal cortex for a representation of the internal and external space (Sereno and Huang, 2014) and this is critical for successful navigation.

Thanks to multisensory integration, it is possible to update the position of the body in the space and to orient ourselves in the environment (Loomis et al., 1993; Thinus-Blanc and Gaunet, 1997; Fetsch et al., 2009; Butler et al., 2010; Prsa et al., 2012; Schmidt et al., 2013).

During navigation, vision is important because it provides information about both the traveler’s motion and the layout of near and far space (Blasch and Welsh, 1980; Foulke, 1982; Strelow, 1985; Loomis et al., 1993). The visual system also provides more accurate information on distance than the auditory system (Loomis et al., 1998). In the absence of vision, such as in blindness, navigation capabilities may result compromised. Some studies support this view, showing impaired skills in blind individuals, associated with inferential navigation (Seemungal et al., 2007) and lower sensitivity to changes in perspective structure when walking without vision (Rieser et al., 1986). They also show slower walking speed, cautious posture, shorter stride length and longer duration of stance compared to sighted and late blind individuals (Nakamura, 1997). On the other hand, some other skills related to spatial navigation are intact even when visual input is missing (for a review Thinus-Blanc and Gaunet, 1997; Seemungal et al., 2007).

During development, vision plays a key role in aligning neural representations of space in the brain (King, 2009, 2014). Multisensory integration areas (including regions underlying audiovisual spatial processing) are significantly reorganized when visual input is absent. Early visual deprivation impacts on the topographic organization of the auditory receptive fields of superior colliculus neurons (King, 2009). Bias in auditory localization has been shown in owls reared with distorting prisms (Knudsen and Knudsen, 1985) and total visual deprivation of young ferrets has been shown to cause disordered development of auditory spatial maps (King and Carlile, 1993). Comparable (but transitory) effects have also been demonstrated in humans (Recanzone, 1998; Zwiers et al., 2003). In the case of blindness, compensatory mechanisms can be adopted to improve spatial skills (Merabet and Pascual-Leone, 2010). This plasticity allows the visual cortex in the congenitally blind individual to become colonized to some extent by the auditory and somatosensory systems (e.g., Sadato et al., 1996; Weeks et al., 2000). As result, a strong and reliable response to sound alone in the primary visual cortex has been observed in blind individuals, using fMRI (Roder et al., 2002; Amedi et al., 2007; Bedny et al., 2011; Lane et al., 2015) and EEG (Kujala et al., 1995; Focker et al., 2012).

Hearing can compensate for the absence of vision by providing inputs for spatial perception in the near and far space because it covers a larger spatial field compared to other senses (Kolarik et al., 2016) and by using allocentric perception of the surrounding space (relative to external objects) rather than egocentric (relative to the observer; Loomis et al., 2001). Psychophysical evidence suggests that some tactile and audio skills in congenital blind individuals result enhanced (e.g., Lessard et al., 1998; Roder et al., 1999; Goldreich and Kanics, 2003; Gougoux et al., 2004; Tinti et al., 2006; Fortin et al., 2008), such as the ability to localize a sound source in the horizontal plane (Lessard et al., 1998; Gougoux et al., 2004; Doucet et al., 2005) or discriminate between different sounds (Gougoux et al., 2004). Studies of animals confirm this view by suggesting that sound processed by neurons in auditory cortical areas can be enhanced following visual deprivation (Korte and Rauschecker, 1993; Petrus et al., 2014).

On the other hand, recent psychophysical works have pointed out that some forms of auditory perception in visually impaired individuals result compromised, raising some doubts about the degree and the limits of cross-modal plasticity in the case of sensory loss. Blind individuals are impaired in complex skills such as sound localization in the mid-sagittal plane (Zwiers et al., 2001; Lewald, 2002; Voss et al., 2015) and tasks requiring a metric representation of the auditory space (Gori et al., 2014; Finocchietti et al., 2015). This impaired audio space perception in blind individuals can hamper the compensation during navigation provided by audition in the absence of vision.

In everyday life, it is fundamental for blind individuals to decode static sounds (e.g., a telephone) and dynamic sounds (e.g., people walking around while speaking), but especially to navigate toward them and engage in interaction. Understanding how static and dynamic sounds are perceived and how they are interpreted during navigation is therefore of fundamental importance. The studies reported above suggest that both impaired auditory representations and impaired navigation strategies might affect everyday interaction of visually impaired individuals. One of the more compromising issues that they encounter is the association between decoded audio signals and navigation strategies to reach the target. What are the internal processes that link auditory perception and navigation in blind individuals? Does past visual experience influence their development? Previous studies have shown that lack of visual experience impacts on navigation skills (Rieser et al., 1986) and on complex auditory perception (Gori et al., 2014; Finocchietti et al., 2015). Starting from this evidence, we hypothesize that the integration between auditory perception and motor responses in blind individuals could be compromised, giving rise to impairments in navigation. Moreover, given that vision is fundamental for space development (King, 2009, 2014), we also hypothesize that prior visual experience should shape this integrative audio-motor process, producing different performances in early and late blind individuals. These two hypotheses were addressed by investigating the relative role of auditory perception, navigation and past visual experience on path reproduction and on path recognition abilities. We evaluated the ability of sighted and blind individuals in recognizing a sonorous stimulus in a localization task involving auditory static sources and dynamic geometrical auditory pathways. To evaluate the ability to reproduce dynamic audio pathways and to reach static sounds we analyzed the movement pattern on both tasks. To evaluate the integration between auditory perception and motor responses we asked participants to report the shape perceived and afterward to reproduce the perceived sonorous pathway by walking. To highlight the role of past visual experience on the development of navigation and auditory perception, we compared the performance of sighted, early blind and late blind participants.

## Materials and Methods

### Participants

Ten visually impaired and 10 sighted individuals participated in the experiment. In the group of blind participants, seven were congenitally blind and three were acquired blind (see **Table 1** for clinical details). All the participants had a similar level of education (at least an Italian high school diploma, indicating 13 years of school). All the early blind participants were blind at birth. None of the participants had any history of hearing impairment. Blind participants were contacted from a list of participants that had taken part in our previous experiments. Sighted participants were contacted from a list of participants that had asked to take part in our studies.

**Table 1:** Clinical details of the early blind (EB) and late blind (LB) participants.

Participant	Age at test	Gender	Pathology	Onset of blindness
**Early blind**				
#EB1	21	F	Glaucoma, retinal detachment	Birth
#EB2	25	F	Retinopathy of prematurity	Birth
#EB3	26	F	Retinopathy of prematurity	Birth
#EB4	36	M	Retinopathy of prematurity	Birth
#EB5	49	M	Retinopathy of prematurity	Birth
#EB6	56	M	Retinopathy of prematurity	Birth
#EB7	56	M	Congenital glaucoma	Birth
**Late blind**				
#LB1	27	M	Corneal opacity	17
#LB2	45	F	Leber amaurosis	40
#LB3	65	M	Glaucoma	14

*The table shows age at the time of testing, gender, pathology, and age at which they became completely blind.*

### Materials and Procedures

**FIGURE 1:**
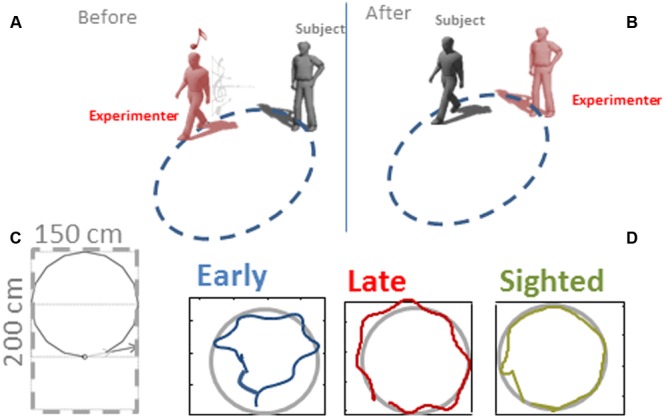
**Representation of the protocol. (A)** Experimenter moved along a path by moving an audio source. **(B)** The participant, after hearing to the audio information, had to reproduce the path. **(C)** The dimension of the surface where the shapes were produced was 200 cm × 150 cm. **(D)** A motion tracking system was used to record the motion trajectories. Three examples trajectories are reported for an early blind, late blind and sighted participant.

**FIGURE 2:**
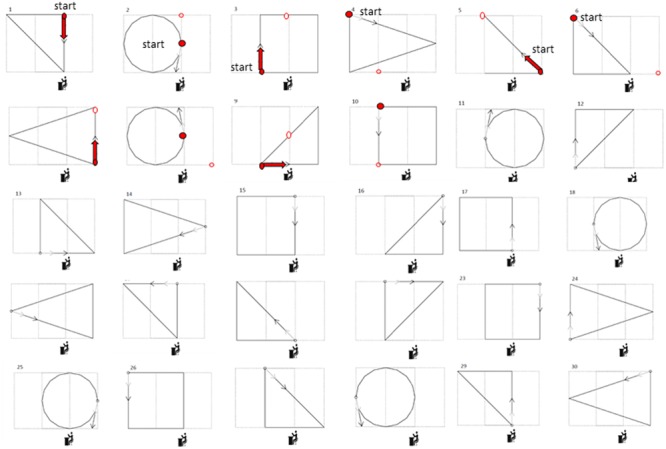
**Complete list of the paths executed.** Thirty shapes were presented during the experiment. After each shape the participant had to reach the next starting position by listening to and reaching a static sound coming from the new position, indicated by the red points in the first 10 shapes. The participant had to move toward the sound source (thus navigating from the circular open to the circular closed red dot) to start the next trial. The icons under each shape represent the position of the second experimenter managing the setup to collect the motion tracking data.

Each participant was asked to reproduce four shapes in different orientations for a total of 30 shape combinations, to categorize all the shapes previously heard, and to walk toward 30 static sound sources, for a total of 90 trials per participant (see **Figures 1** and **2**). The four shapes considered in the study were a square with an area of 4 m^2^, a triangle with an area of 2 m^2^, a triangle with an area of 3 m^2^, and a circle with an area of 3.14 m^2^. The sound was a single pulse at 500 Hz, intermittent sound at 180 bpm, as previously used by our group (Finocchietti et al., 2015). The experiment was performed in a dark room, with low light allowing the experimenter to see, but at the same time not perceivable by the blindfolded participants. When the participants arrived, we briefly outlined the experiment and they were asked to read and sign the written informed consent form. For the blind participants, the ethical documentation was read by the experimenter. Before the testing, all participants were blindfolded, guided into a motion recording room and positioned at the starting point. The room had a rectangular floor (300 × 200 cm) that was defined by the recording space that our motion tracking system could cover (Vicon Motion Systems Ltd. UK). Ten different landmarks and relative connections between each other were marked with colored tape on the floor (**Figures 1** and **2**). A spherical marker was mounted on a hat positioned on the head of both the participant and the experimenter for motion tracking. In addition to the marker, the experimenter also had an audio source positioned in the hat. Three experimenters instructed the participant and performed all the experiments (MG, GC, and SF). The experimenters had been previously trained to perform the task as uniformly as possible, so that the movement velocity was consistent across trials, positions, and groups. The participants were randomly assigned to one of the three experimenters. The participant was positioned at the starting point, corresponding to the initial position of the first shape (**Figure 2** on the top left, shape 1). Each trial was structured in three phases as follow:

#### Phase 1

The experimenter navigated following the first shape reported in **Figure 2**. After having heard the audio motion (**Figure 1A**), the participant had to report which shape was produced by the experimenter. The participant knew in advance that the navigation could follow a square, circular, or triangular path.

#### Phase 2

After having reported the shape perceived, the participant had to navigate to reproduce the path previously heard and return to the initial position (**Figure 1B**). The participant was always positioned facing the first motion direction of the geometric shape.

#### Phase 3

The experimenter moved toward a new starting position (reported in **Figure 2** as the starting point in the second circular shape, red dot) and produced a 1 s static sound. The participant had to move toward the sound source (thus navigating from the circular open to the circular closed red dot) to start the next trial. In this phase the participant was not oriented by the experimenter toward the static sound but had to orient himself/herself by rotating his/her body and then walking, starting from the final orientation and position reached with the previous navigation trial. All the shapes in **Figure 2** were reproduced in the same order. The static sounds to be reached have been reported for the first 10 shapes. Before starting a new trial, the participant was always oriented frontally toward the direction of the first segment of the shape (indicated by the arrow in **Figure 2**).

### Data Analysis

**FIGURE 3:**
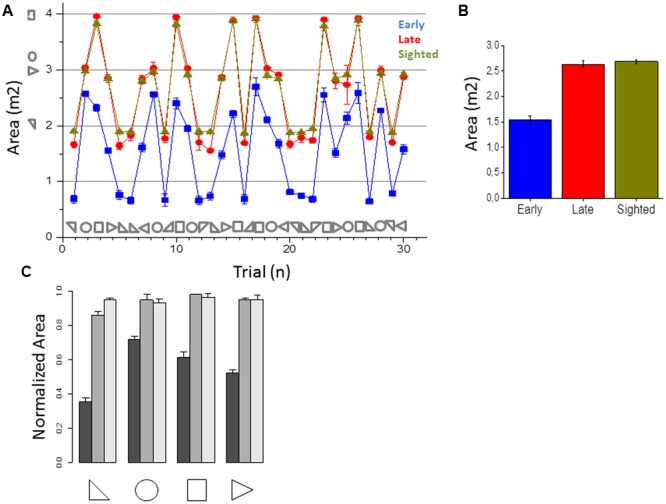
**Area of the shapes reproduced. (A)** Average area reproduced for early blind (in blue), late blind (in red), and sighted participants (in green) for all the shapes considered. **(B)** Average of the areas reproduced, calculated by merging all the 30 shapes for each participant group. Correct area for each shape is reported with symbols on the abscissa. **(C)** Normalized area (mean ± SD) for early blind (dark gray), late blind (gray), and sighted participants (light gray) for all the shapes considered.

**FIGURE 4:**
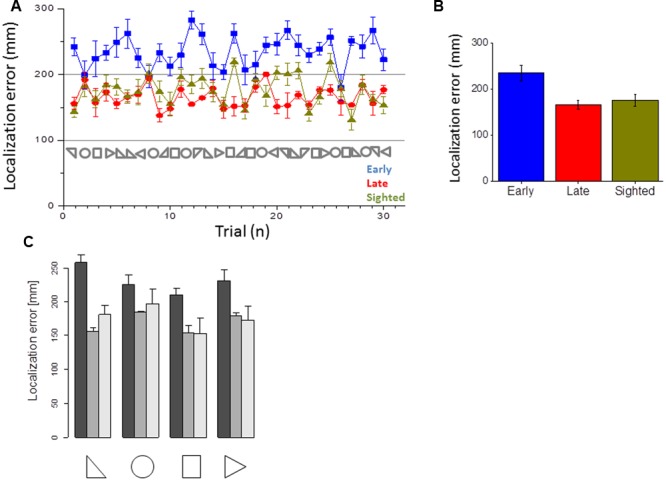
**Error in closing the shape. (A)** Average error made in the closure of the shape for early blind (in blue), late blind (in red), and sighted participants (in green) for all the shapes considered. **(B)** Average errors made, calculated by merging all the 30 shapes for each participant group. **(C)** Average of the errors made, for the three groups and the four categories of shapes.

**FIGURE 5:**
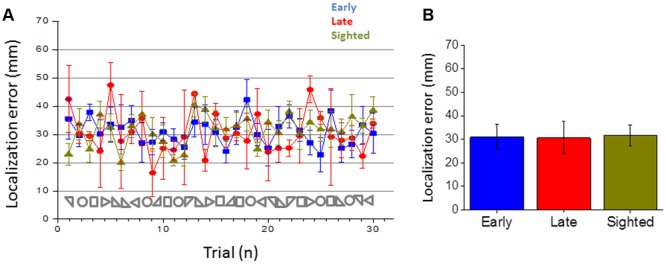
**Error in reaching the static point. (A)** Average error made in reaching the static point for early blind (in blue), late blind (in red), and sighted participants (in green) for all the shapes considered. **(B)** Average errors made, calculated by merging all the 30 shapes for each participant group.

Kinematic data were post-processed and analyzed using Matlab (R2013a, The MathWorks, USA). Spatial accuracy and localization error during navigation was computed for each participant and for each spatial position (see **Figure 1B** as example of motion tracks for three participants). The area of the shape produced was computed by considering the limits of the trajectories performed by the participant. The end-point was calculated for both shape reproduction and static sound reaching. Each end-point position (x_pos, y_pos) was computed as the average of the last 10 samples and normalized on the final position (the end of the shape or the end of the linear trajectory toward the static sound), in order to avoid movement errors. Area values were averaged based on the number of participants for each of the 30 shapes and normalized by dividing it by the actual shape area (**Figure 3**). For each shape, the localization error was calculated as the Euclidean distance (in mm) between the position reached by the participant at the end of reproduction and the correct final position reached by the experimenter (**Figure 4**). For the static sound, the localization error was calculated as the Euclidean distance (in mm) between the final position reached by the participant and the position where the static sound source was positioned (**Figure 5**). Correct sound localization was defined as the difference from the experimenter and participant categorization was used for further analysis. Shape recognition data were recorded by collecting the verbal responses of the participants. Data were normally distributed, confirmed by visual inspection of Q–Q plots. Data are presented as mean and standard error (SE). Absolute area and normalized area and localization error for shape reproduction trials were analyzed with three repeated-measure ANOVA with shape (circle, square, triangle1, triangle2) as within-participant factor and group (EB, LB, sighted) as between-participant factor. The localization error for static sound was analyzed with a one-way ANOVA with group (EB, LB, sighted) as factor. The percentage of correct responses and velocity of movement were analyzed with a one-way ANOVA with group (EB, LB, sighted) as factor. Olejnik and Algina (2003) generalized Eta (η_G_) was used to compute effect size. Tukey HSD was used to test significance of multiple comparison *post hoc* tests. Data from late blind individuals, even if only collected in three subjects, were analyzed separately from sighted and early blind to show a specific role of visual experience on this task. Visual inspection of the distribution of the residuals against the fitted values and of quantile–quantile plots indicated that the basic requirements of ANOVAs (homoscedasticity and normality) were satisfied.

## Results

### Navigation Responses

This section reports the results of the navigation skills of early blind, late blind and sighted individuals in reproducing the audio shapes and in reaching static sounds. **Figure 3A** reports the areas for all the shapes and **Figure 3B** the average area reported for the three groups. Sighted and late participants (in yellow and red) performed the task correctly while early blind participants (in blue) show a general compression of the surrounding space (correct area for each shape is reported with symbols on the abscissa). Statistical analysis (ANOVA) revealed a very large group effect [*F*(2,17) = 2701.4, *p* < 0.01, η_G_ = 0.89]. The shape effect and interaction were also statistically significant but had a much smaller effect [*F*(3,51) = 137.9, *p* < 0.01, η_G_ = 0.06 and *F*(6,51) = 104.4, *p* < 0.01, η_G_ = 0.10, respectively]. On average, the area of the shapes reproduced by the EB was 55% the size of the reference area (**Figure 3C**). In contrast, the control and LB groups reproduced shapes that were only slightly smaller than the reference shapes (95 and 93%, respectively, **Figure 3C**). Tukey HSD *post hoc* analyses between the group levels for significance indicated that the average area reported between early blind participants were significantly lower (in blue, *M* = 1.54, *SE* = 0.07) than that one of the late blind (in yellow, *M* = 2.6, *SE* = 0.06, *p* < 0.001) and sighted group (in red, *M* = 2.68, *SE* = 0.04, *p* < 0.001), which did not differ statistically from each other. These results suggest that early blind individuals show some navigation impairments compared with sighted and late blind individuals when they have to reproduce a previously heard shape but not when they have to reach a static sound. Furthermore, the localization error to close the shape (i.e., on reaching the initial starting point) was higher for all the shapes in early blind than in sighted and late blind individuals (**Figure 4**). **Figure 4A** reports the error in closing the shapes created. This error represents the difficulty of localizing the initial starting point. On average (**Figure 4B**), sighted and late blind individuals (in yellow and in red) are also significantly more precise in this task compared to early blind individuals (in blue). Statistical analysis (ANOVA) showed a Main effect for participant group [*F*(2, 17) = 65.409, *p* = 0.001, η_G_ = 0.69]. In early blind individuals the error to close the shape is higher for all the shapes considered (**Figure 4C**). Again, both the shape and interaction are statistically significant but the effect size much smaller [Shape: *F*(3,51) = 11.715, *p* < 0.001, η_G_ = 0.09; Group × Shape interaction: *F*(6,51) = 5.10, *p* < 0.001, η_G_ = 0.08]. *Post hoc* tests (Tukey HSD) also indicated that the early blind group (*M* = 234.9, *SE* = 16.2) differed from the late blind group (*M* = 165.7, *SE* = 9.8, *p* < 0.001) and the sighted group (*M* = 176.6, *SE* = 13, *p* < 0.001). **Figure 5** represents the error associated with the localization of static audio sources. Interestingly, early blind individuals showed no deficit in reaching static sounds (**Figure 5**). In contrast to the shape reproduction task, all participants localized the static audio information in a similar way (**Figures 5A,B**) [one-way ANOVA, *F*(2,77) = 0.206, *p* = 0.815]. These results suggest a navigation problem in early blind individuals only when they are asked to reproduce an auditory geometric path, not when they are asked to reach a static audio source.

### Audio Shape Recognition

**FIGURE 6:**
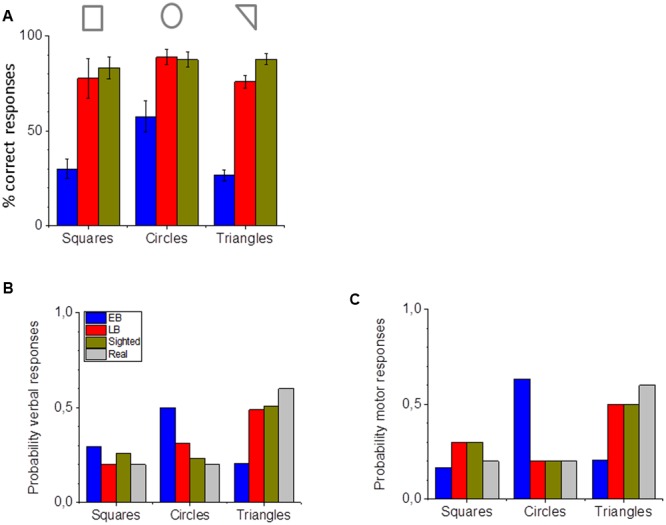
**Shape perception and reproduction. (A)** Percentage of correct verbal responses for the three categories of shapes used for early blind (in blue), late blind (in red), and sighted participants (in green). **(B)** Probability of identifying the correct shape with verbal responses for early blind (in blue), late blind (in red), and sighted participants (in green). **(C)** Probability of reproducing the correct shape with motor navigation for early blind (in blue), late blind (in red), and sighted participants (in green). The actual probability of the shape occurring is presented in **(B)** and **(C)** in gray.

This section reports the results of geometrical auditory shape recognition and shape motor reproduction. **Figure 6A** reports the percentage of correct responses for the square, circular, and triangular shapes listened. Early blind individuals fail to correctly perceive the shapes, especially triangles and squares, for which barely 30% of responses were correct [**Figure 6A**, one-way ANOVA, *F*(2,17) = 126.4933, *p* < 0.001]. A Tukey *post hoc* test revealed that it was statistically significantly higher for the early blind group (Squares: *M* = 30, *SE* = 5.8; Circles: *M* = 57.6, *SE* = 4; Triangles: *M* = 26.62, *SE* = 3.1) compared to the late blind (Squares: *M* = 77.8, *SE* = 5.2; Circles: *M* = 88.8, *SE* = 8; Triangles: *M* = 75.9, *SE* = 2.7, *p* < 0.001) and the sighted group (Squares: *M* = 83.3, *SE* = 10.4; Circles: *M* = 87.5, *SE* = 4; Triangles: *M* = 87.9, *SE* = 3.4, *p* < 0.001). **Figure 6B** reports the probability of perceiving the three categories of shapes and **Figure 6C** indicates the probability of reproducing the three categories of shapes for the three groups of participants, compared with the real probability that the specific shape occurred (in gray). The primarily motor response of early blind individuals is the circle, with a probability of over 0.6.

While sighted and late blind individuals are very good at recognizing all the different shapes (**Figure 6**), early blind individuals’ performance is worse for all the three shapes considered. The evidence that they better recognize the circles (**Figure 6A**) is probably due only to their tendency to frequently report the circle shape (**Figure 6B**). Interestingly, if we compare their perceptual response (**Figure 6B**) and their navigation reproduction (**Figure 6C**) we can observe a similar pattern. Contrarily to sighted (in yellow) and late blind (in red) individuals, who perceived and reproduced all the shapes correctly (gray bar), early blind (blue) individuals tended to report and to reproduce more circles. Results suggest that the probability of perceiving circles was different among the three groups for the perceptual circular responses [**Figure 6B**, one-way ANOVA, *F*(2.17) = 104.18615, *p* < 0.001] and motor circular responses [**Figure 6C**, one-way ANOVA, *F*(2.17) = 126.4933, *p* < 0.001]. A Tukey *post hoc* test (Tukey HSD) revealed that it was statistically significantly higher for the early blind compared to the late blind (*p* < 0.001) and to the sighted group (*p* < 0.001) for both perceptual and motor tasks.

### Perceptual vs. Navigation Response

**FIGURE 7:**
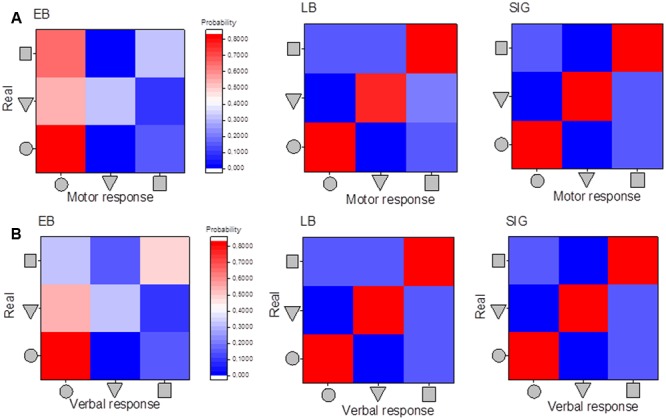
**Matrices of confusion: association between motor and perceptual responses. (A)** Matrices of confusion for the motor responses and the three categories of shapes used. **(B)** Matrices of confusion for the perceptual responses and the three categories of shapes used. Responses of early blind participants are reported in the first column, of late blind in the second column, and of sighted in the last column. The level of probability is associated with the colored scale presented: red means high probability and blue low probability of reporting the specific shape, middle colors indicate intermediate probability.

This section reports the results on associating perceptual and motor responses in the three groups. To evaluate this, we reported in **Figure 7** the matrices of confusion for both the perceptual task (upper line) and the motor task (lower panel). While identical patterns can be observed for sighted and late blind individuals in both tasks, early blind individuals show a different pattern of perceptual and motor responses, suggesting a mismatch between perceptual audio shape recognition and navigation shape reproduction. In late blind and sighted individuals, responses show a red equality line, suggesting that the shapes reported and reproduced are the same as the shape presented. The results of early blind individuals did not show the red equality line, but there were more responses associated with the circular shape in both the perceptual and the motor responses. This is particularly true for the square shape (**Figure 7**, left column). Early blind individuals are more likely to report perceiving a square when they actually hear a square. On the other hand, although they intended to reproduce a square, they actually reproduced a circular shape (see the first column of the matrix). This mismatch points to a third problematic aspect, i.e., the inability of early blind individuals to navigate and reproduce the desired path.

**FIGURE 8:**
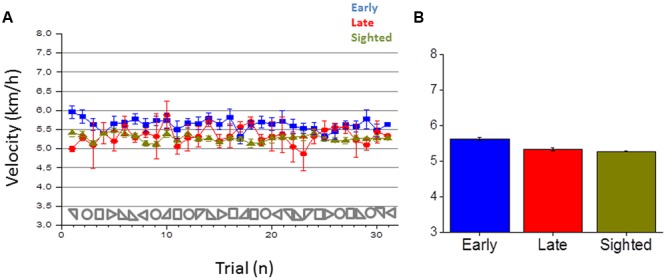
**Velocity in reproducing the shape. (A)** Average velocity in reproducing a shape for early blind (in blue), late blind (in red), and sighted participants (in green) for all the shapes considered. **(B)** Average velocity, calculated by merging all the 30 shapes for each participant group.

To highlight possible differences among the three groups, we also report in **Figure 8A** the walking speed of participants for all the different shapes and groups. **Figure 8B** reports the average velocity for the three groups. Results suggest that walking speed was different among the three groups [one-way ANOVA, *F*(2.17) = 59.00275, *p* < 0.001]. A Bonferroni *post hoc* test revealed that the walking speed to reproduce a shape was statistically significantly higher for the early blind (*M* = 5.6, *SE* = 0.04) compared to the late blind (*M* = 5.3, *SE* = 0.04, *p* < 0.001) and to the sighted group (*M* = 5.3, *SE* = 0.01, *p* < 0.001). The speed of the sighted and late blind group was not significantly different (*p* = 0.78). The different walking speed among groups could be another aspect that might negatively affect their navigation capabilities.

## Discussion

In this work, we have investigated how spatial representation of the environment is shaped by sensory experience by studying cross-modal plasticity mechanisms in early and late blind individuals during navigation tasks. The lack of visual experience in blind individuals impacts on navigation skills (Rieser et al., 1986) and on complex auditory perception (Gori et al., 2014; Finocchietti et al., 2015). Starting from this evidence, we hypothesize that integration of auditory and motor responses could be compromised in blind individuals. This could give rise to impairments in navigation. Since vision is fundamental for space development (King, 2009, 2014), we also hypothesize that visual experience in the first period of life could be important for audio-motor integration development. If this is true then a different performance in early and late blind individuals should be observed. Both hypotheses have been confirmed by our study. Our first hypothesis was supported by audio shape recognition and navigation deficits observed in early blind participants. Our second hypothesis was supported by the fact that the deficit was specific for early and not late blind individuals, who perform all the tasks in a similar way to sighted individuals. In general, our study indicates that plasticity mechanisms in blind individuals might not fully compensate for lack of vision: some sensory-motor skills not directly dependent on visual experience are correctly acquired by blind individuals; some other sensory-motor skills directly dependent on visual experience are compromised, probably because their acquisition requires the development of dedicated neural networks which rely on visual input.

### Audio Shape Recognition

In support of our first hypothesis, firstly, we observed a deficitary pattern of auditory spatial analysis or representation of dynamic auditory geometric cues for spatial navigation in early blind participants. Previous works, however, have shown superior processing of audio motion (e.g., Lewald, 2013; Jiang et al., 2016) for artificially moving stimuli positioned in front of early blind individuals. We think that the performance observed in our auditory task can be explained with two main considerations: Firstly, the stimulus presented in our study needed to be integrated for longer spatial and temporal windows than the stimuli used in previous experiments. Secondly, the task required the participant to build a complex metric representation of the space by memorizing and comparing the segments presented in each geometric figure. Both these aspects may require an Euclidian representation of space that has been shown to be compromised in blind individuals (Gori et al., 2014; Finocchietti et al., 2015; Gori, 2015; Vercillo et al., 2015, 2016). Finally, another aspect to be considered is that the task required the participant to construct a spatial representation not only by discriminating basic directional changes (left and right) but also by sequentially updating angular patterns derived from the shape. Early blind individuals could have a deficit in recognizing changes in audio movement direction. Early blind individuals tend to perceive and reproduce a circular path even when presented with angular shapes. The circular is represented with a continuous sound without the pauses associated with angular paths. More studies would be necessary to clarify the difficulty of early blind individuals in processing changes in audio movement direction. As previously mentioned, vision plays a dominant role in aligning neural maps of space in the midbrain during development (King, 2009). Auditory maps are usually shaped to match the visual field representation in the superior colliculus (King, 2014). The deficit observed here could be attributed to the lack of visual input on the natural integration process that permits us to associate signals provided by the eyes and by the ears about a common stimulus source. Interestingly, our results suggest that the lack of such refined maps does not compromise the ability to correctly perform activities related to spatial skills such as the localization of static sounds in space. Indeed, early blind participants performed as well as sighted individuals and late blind individuals in the localization of static sound sources. This result is in accordance with the idea that compensatory processes can be present in the absence of vision and that cortical reorganization may enhance some kind of auditory spatial performance in blind individuals (Collignon et al., 2009). It is also in accordance with other previous works (Ashmead et al., 1989; Lessard et al., 1998; Roder et al., 1999; Lewald, 2002, 2013; Voss et al., 2004; Kolarik et al., 2013) which suggest that blind individuals have normal or supra-normal sound localization abilities compared to sighted participants (Collignon et al., 2009; Voss et al., 2015) and that they show more accurate information with regard to distance (Zahorik, 2001; Kolarik et al., 2013, 2016).

### Navigation Responses

Secondly, we observed a different navigation pattern in early blind individuals: (i) they compress the shapes to be reproduced during navigation, (ii) they tend to reproduce more circular paths and, (iii) they tend to move faster. We think that all these aspects can be associated with an egocentric frame of reference adopted by blind individuals during navigation. Spatial navigation can be differentiated in route navigation, that refers to egocentric coordinates, and in inferential navigation that relies on allocentric coordinates (Loomis et al., 1993; Thinus-Blanc and Gaunet, 1997; Schmidt et al., 2013). Visual information in the first period of life may provide a contextual frame of reference to encode stimuli using allocentric (relative to external objects) rather than egocentric (relative to the observer) coordinates (Pasqualotto and Proulx, 2012). Some studies support this view, showing that blind individuals rely more on route navigation which is based on kinematic strategies relative to the experienced movement by using an egocentric reference (Bigelow, 1992). They encode stimuli using allocentric rather than egocentric, coordinates (Roder et al., 2007, 2004; Pasqualotto and Proulx, 2012). Impaired abilities in blind individuals associated with inferential navigation (Seemungal et al., 2007) and lower sensitivity to changes in perspective structure when walking without vision (Rieser et al., 1986) have been also reported. We think that spatial impairments delineated in this work can be associated with the use of a route navigation strategy by early blind individuals. This could indeed explain why they tend to be more likely to perform circular paths than angular paths. To perform a circular path they can rely on vestibular and proprioceptive information that is not impaired (Valko et al., 2012; Moser et al., 2015): they can internally set a specific rotation, speed and acceleration and maintain it during the entire path. Contrarily, in the square and triangular shapes, early blind participants need to set a metric between the geometrical components and to segment the movement with many stop-rotate and go sub-tasks. These aspects might affect navigation and orientation, especially for the reproduction of triangular and square shapes, where the body representation in the space and body coordination is more important. This might require a more complex spatial representation for which visual experience is fundamental. The use of a route navigation strategy is also supported by the different walking speed we observed in early blind individuals when compared with sighted and late blind individuals. Early blind individuals walk faster than the other two groups: in sighted individuals the slower walking speed could be associated with high task complexity, energy requirements and equilibrium demands in an unusual non-visual navigation task. Following the same line, the higher velocity in early blind individuals can be attributed to familiarity with non-visual tasks. On the other hand, the fact that late blind individuals also show slower walking speed compared to early blind participants, suggests that the walking pattern might be associated with early visual experience more than with confidence with the task. A possible explanation, which is in accordance with our previous observations, is that slower walking speed could facilitate spatial perception in an allocentric frame of reference where spatial-temporal information needs to be integrated in order to comprehend the global path produced. Contrarily, a faster walking speed, observed in sighted and late blind individuals, could reflect a more egocentric frame of reference in which global information is not integrated in space and time. Finally, we can speculate that the shape compression around the body region observed in early blind individuals can similarly reflect a more egocentric frame of reference that attracts the navigation toward the initial body position, resulting in it being compressed around the body. Further studies will be necessary to clarify this aspect.

### Visual Experience

From our results a difference between early and late blind individuals clearly emerges, supporting our second hypothesis. Early, but not late blind or sighted, participants had difficulties in performing the auditory and navigation tasks: the impairment was specific for complex audio paths, suggesting that simpler tasks such as static audio localization might require less subtle mechanisms. The performance of late blind and sighted individuals is similar in that they do not compress the area when reproducing the path and they correctly and precisely find the end point of the shape performed. Although only few late blind individuals were tested the result is stable and the variability among participant was minimal. This is in accordance with previous studies showing that the age of onset and duration of sight loss can affect auditory abilities (Voss et al., 2004; Gori et al., 2010; Wan et al., 2010) and the extent of cross-modal recruitment in dorsal brain regions in response to auditory spatial information (Dormal et al., 2012).

## Conclusion

To conclude, our results suggest that early blind individuals have a significant deficit in the interpretation of auditory geometric cues for navigation. The present study provides support for the cross-sensory calibration theory (Gori et al., 2008; Burr and Gori, 2011; Burr et al., 2011; Gori, 2015), suggesting that visual information is necessary in the first period of life for the normal development of auditory spatial representations. The auditoryimpairment observed in this study could be related to the lack of Euclidean representation which is typically mediated by the visual modality in sighted individuals. The navigation impairment observed in this study, on the other hand, may be related to the persistence of an egocentric frame of reference in early blind individuals. We can speculate that the visual cortex plays a key role in the transformation from egocentric to allocentric reference systems and that coordinate transformation could be mediated in sighted individuals by pathways involving the superior colliculus as previously proposed (King et al., 1988; Gori et al., 2014; King, 2014). We can also speculate that the lack of allocentric representation might hamper the processing of complex auditory geometric cues for spatial navigation investigated in this work. This could result from a different construction of the topographical representation of auditory space and suggests that the role of vision in this transformation process could occur in the first years of development, since the deficit is not present in late individuals.

Efficient walking is mediated by the integration of audio-tactile signals with motor feedback. Understanding how this process occurs in early blind individuals might improve the development of scientific driven rehabilitation technologies for navigation. We hope that this work can provide inputs for further studies to better understand how non-visual navigation can be optimized in visually impaired individuals and what are the limits of cortical plasticity in case of sensory loss are.

## Ethics Statement

The study was approved by the ethics committee of the local health service (Comitato Etico, ASL3 Genovese, Italy). All the participants had a similar level of education (at least an Italian high school diploma, indicating 13 years of school). All the early blind participants were blind at birth. None of the participants had any history of hearing impairment. Blind participants were contacted from a list of participants that had taken part in our previous experiments. Sighted participants were contacted from a list of participants that had asked to take part in our studies. When the subjects arrived, we briefly outlined the experiment and they had to read and sign the ethical approval form. For the blind participants, the ethical documentation was read by the experimenter. All the participants provided written informed consent in accordance with the Declaration of Helsinki.

## Author Contributions

MG, SF, and GC collected data. SF and MG analyzed the data. MG wrote the manuscript. SF, GC, and GB-B participated in protocol definition and paper discussion.

## Conflict of Interest Statement

The authors declare that the research was conducted in the absence of any commercial or financial relationships that could be construed as a potential conflict of interest.
