# Corrigendum: Shape Perception and Navigation in Blind Adults

**DOI:** 10.3389/fpsyg.2017.00882

**Published:** 2017-06-12

**Authors:** Monica Gori, Giulia Cappagli, Gabriel Baud-Bovy, Sara Finocchietti

**Affiliations:** ^1^Unit for Visually Impaired People, Istituto Italiano di TecnologiaGenoa, Italy; ^2^Robotics, Brain and Cognitive Science Department, Istituto Italiano di TecnologiaGenoa, Italy; ^3^The Unit of Experimental Psychology, Division of Neuroscience, IRCCS San Raffaele Scientific Institute, Vita-Salute San Raffaele UniversityMilan, Italy

**Keywords:** shape, perception, audio perception, blindness, motor, navigation

There is an error in the Funding statement. The correct Name for the Funder is **This research has been supported by the European ABBI project (FP7-ICT 611452)**. The authors apologize for this error and state that this does not change the scientific conclusions of the article in any way.

## Conflict of interest statement

The authors declare that the research was conducted in the absence of any commercial or financial relationships that could be construed as a potential conflict of interest.

